# Associations between overweight/obesity and increased levels of serum inflammatory markers from prodromal stages to chronic psychosis

**DOI:** 10.1192/j.eurpsy.2023.815

**Published:** 2023-07-19

**Authors:** A. Michalczyk, P. Podwalski, K. Waszczuk, E. Tyburski, E. Cecerska-Heryć, J. Samochowiec

**Affiliations:** 1 Department of Psychiatry; 2 Department of Health Psychology; 3 Department of Laboratory Medicine, Pomeranian Medical University, Szczecin, Poland

## Abstract

**Introduction:**

Chronic subclinical inflammation is considered to be an important contributor to the development of schizophrenia. Meta-analyses confirm the presence of higher levels of inflammatory markers in schizophrenia and its prodromal stages compared to controls, however studies differ in terms of associated cytokines. Obesity is a common problem in patients with schizophrenia and, at the same time, it is recognized as a source of subclinical inflammation in the general population.

**Objectives:**

The aim of the study was to verify if there is an association between the presence of overweight/obesity and higher levels of CRP and IL-6 in various stages of psychotic disorders and if these factors may influence the course of the disease

**Methods:**

Study was performed in four study groups: 31 healthy controls (HC), 16 patients with ultra-high risk of psychosis (UHR), 30 with first episode of psychosis (FEP) and 71 with chronic schizophrenia (SCH). The severity of psychopathological symptoms in SCH was assed using the Positive and Negative Syndrome Scale (PANSS). The serum levels of inflammatory markers were measured using sensitive ELISA tests.

**Results:**

Study groups significantly differed in the levels of CRP and IL-6. The presence of overweight/obesity was associated with significantly higher levels of CRP in CON and UHR and IL-6 in CON, FEP and SCH. IL-6 was positively correlated with the severity of positive symptoms in PANSS in SCH, however neither IM or BMI were associated with other psychopathological symptoms or number and frequency of exacerbations in schizophrenia patients.

**Conclusions:**

Overweight/obesity is associated with subclinical inflammation in both healthy controls and patients with various stages of psychotic disorders. Subclinical inflammation may be correlated with the course of the disease, however we did not find any direct associations between overweight/obesity and the severity of symptoms. Further studies are needed to verify, if reduction of BMI would be beneficial in reducing levels of inflammatory markers and alleviating disease course.

This research was funded by the Polish Minister of Science and Higher Education’s program named “Regional Initiative of Excellence” in 2019–2022, grant number 002/RID/2018/2019 to the amount of 12 000 000 PLN, and the National Science Centre, Poland, grant number 2019/03/X/NZ5/00719.

**Disclosure of Interest:**

None Declared

